# Supplementary material for: Transcriptome analysis of Tamarix ramosissima leaves in response to NaCl stress
Source: PLoS One. 2022 Mar 31;17(3):e0265653. doi: 10.1371/journal.pone.0265653 (PMC8970367; doi:10.1371/journal.pone.0265653)
Supplement: S1 Fig — (PDF) [file pone.0265653.s001.pdf]

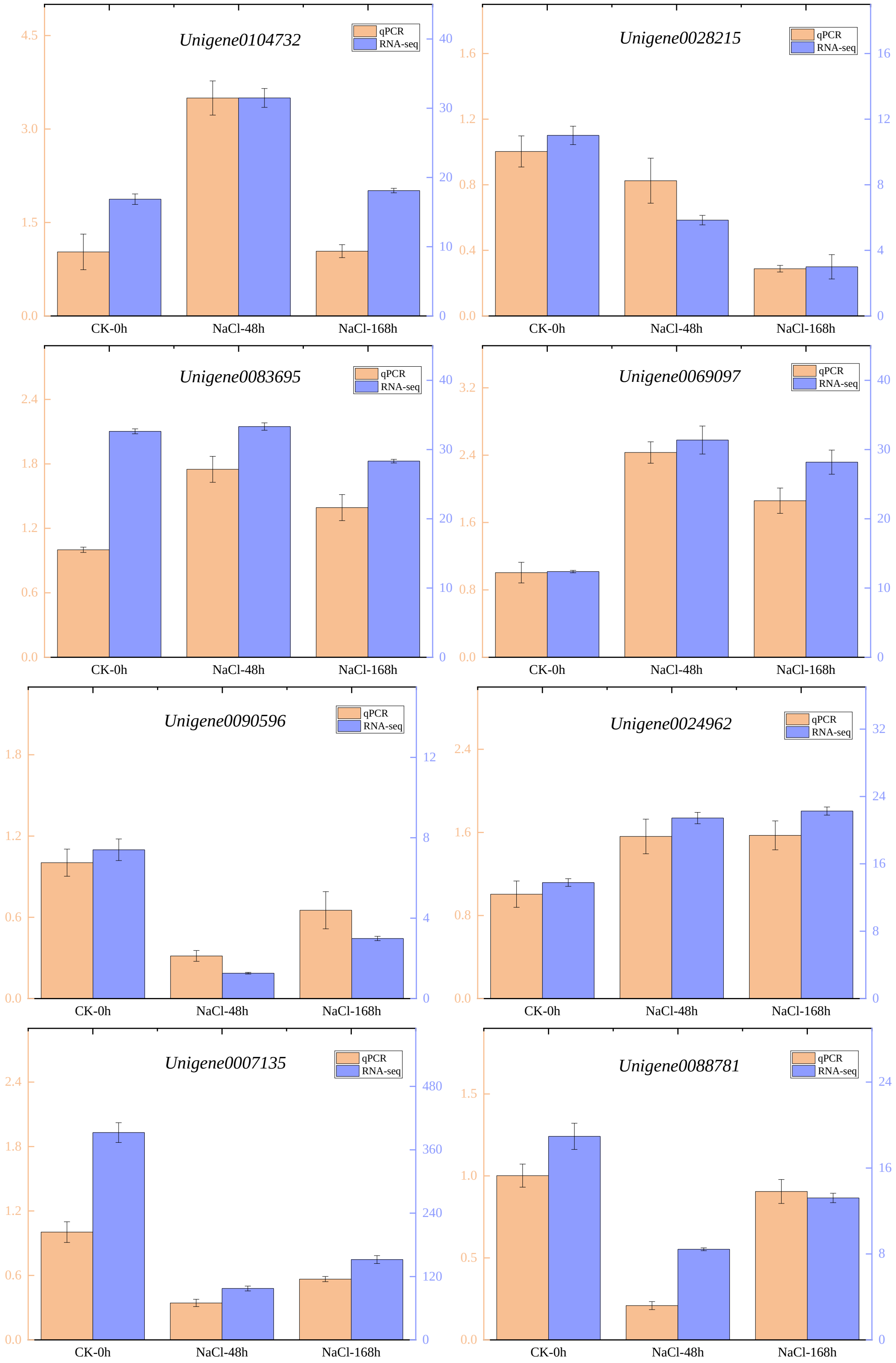

Supplemental Fig.1 Validation of DEGs by qRT-PCR

(Eight differentially expressed genes were randomly selected for qRT-PCR validation, The error bars were obtained from multiple replicates of qRT-PCR.)
