# Supplementary material for: Transcriptome analysis of Tamarix ramosissima leaves in response to NaCl stress
Source: PLoS One. 2022 Mar 31;17(3):e0265653. doi: 10.1371/journal.pone.0265653 (PMC8970367; doi:10.1371/journal.pone.0265653)
Supplement: S1 Table — (PDF) [file pone.0265653.s003.pdf]

Supplemental Table.1 Sequences of specific primers

Primer name	Primer sequence (5'-3')
Unigene0104732	F:TGGCCGGTCCACCCGTATCC<br>R:GCTGACAACCGAACGGCGGA
Unigene0028215	F:CGGTGGCGCAAGGAGCTGTT<br>R:CATCACCACCGCCACCGACC
Unigene0083695	F:ACCCTGCGCCCATCCCTCTT<br>R:CGGCGGAGGGCCGAGTTTAT
Unigene0069097	F:AGCAGCCGATTGTCTCCTTGGGA<br>R:GCACTGCTCCTTTCTCCCTCTGC
Unigene0090596	F:TCCCGCAGTACCTGCTCACGA<br>R:TGGAGACCCCGACGAGGTGG
Unigene0024962	F:ACCATGTCGGCCCGCTTGAC<br>R:TCCGCTGCAGTGGCCCTAGT
Unigene0007135	F:AGGAAGGCGGTGAGGGTGCT<br>R:GCAGCACCGGGAGTCGTAGC
Unigene0088781	F:GGTGGTGGCGGCGGTGATAC<br>R:TGCTGCAACTGCCGCTCCTC
Actin	F:TCGTAGCAGAGCATCGGAGAA<br>R:TGACCCATGCCAACCATAACA
