# Supplementary material for: Transcriptome analysis of Tamarix ramosissima leaves in response to NaCl stress
Source: PLoS One. 2022 Mar 31;17(3):e0265653. doi: 10.1371/journal.pone.0265653 (PMC8970367; doi:10.1371/journal.pone.0265653)
Supplement: S2 Table — (PDF) [file pone.0265653.s004.pdf]

Supplemental Table.2 Randomly select 8 differentially expressed genes

Gene ID	Description
Unigene0104732	Transcription factor bHLH48-like
Unigene0028215	Transcription factor bHLH112 isoform X1
Unigene0083695	bZIP transcription factor 44-like
Unigene0069097	Vacuolar membrane Na+/H+ antiporter
Unigene0090596	Sodium transporter HKT1
Unigene0024962	WRKY transcription factor 1
Unigene0007135	WRKY33-1
Unigene0088781	Transcription factor MYB4-like
