# Supplementary material for: Transcriptome analysis of Tamarix ramosissima leaves in response to NaCl stress
Source: PLoS One. 2022 Mar 31;17(3):e0265653. doi: 10.1371/journal.pone.0265653 (PMC8970367; doi:10.1371/journal.pone.0265653)
Supplement: Attachment — Submitted filename: Plos One.pdf [file pone.0265653.s005.pdf]

PLOS ONE

Transcriptome analysis of Tamarix ramosissima leaves in response to NaCl stress

--Manuscript Draft--

**Manuscript Number:**	PONE-D-21-38993
**Article Type:**	Research Article
**Full Title:**	Transcriptome analysis of Tamarix ramosissima leaves in response to NaCl stress
**Short Title:**	Transcriptome analysis of Tamarix ramosissima leaves in response to NaCl stress
**Corresponding Author:**	Ning Zhang<br>The University of British Columbia<br>Vancouver, British Columbia CANADA
**Keywords:**	T. ramosissima; NaCl stress; Transcriptome sequencing; Transcription factor; ROS scavenging mechanisms
**Abstract:**	Abstract: Halophyte Tamarix ramosissima. Lcdcb (T. ramosissima) are known as the representative of Tamarix plants that are widely planted in salinized soil. However, molecular mechnisms towards salt tolerance and adaptation are largely rare in T. ramosissima. In this study, we carried out RNA-sequence and transcriptome analysis of T. ramosissima in response to NaCl stress, and screened differentially expressed genes (DEGs) and further verified by qRT-PCR. Rsults showed that 105702 unigenes were spliced from the raw data of transcriptome sequencing, where 54238 unigenes were retrieved from KEGG, KOG, NR, and Swiss-Prot, and the remaining 51464 unigenes could not be annotated temporarily. After 48 hours of NaCl treatment, the expression levels of 6374 genes in T. ramosissima leaves were increased and 5380 genes were decreased. After 168 hours of NaCl stress treatment, the expression levels of 3837 genes were up-regulated and 7808 genes were down-regulated. In particular, 8 highly differentially expressed genes (DEGs) were obtained, which mainly encode WRKY, MYB, and bZIP family transcription factors. In addition, the analysis of the KEGG pathway shows that under NaCl stress, a total of 39 genes in T. ramosissima leaves have significant changes in the expression of ROS scavenging mechanisms. After 48 hours of NaCl stress treatment, 21 genes were up-regulated and 18 genes were down-regulated as well as 15 genes were up-regulated and 24 genes were down-regulated after 168h. T. ramosissima may adapt to salt stress by enhancing the ROS scavenging mechanism, including SOD, POD, CAT, APX, GPX, GST, and GR. In this study, WRKY, MYB, and bZIP, and other transcription factors may participate in the process of salt tolerance regulation in T. ramosissima under NaCl treatment. This study provides gene resources and theoretical basis for further molecular mechanisms of salt tolerance in T. ramosissima.
**Order of Authors:**	Yahui Chen
	Guangyu Wang
	Hongxia Zhang
	Ning Zhang
	Jiang Jiang
	Zhizhong Song
**Additional Information:**	
**Question**	**Response**
**Financial Disclosure**<br><br>Enter a financial disclosure statement that describes the sources of funding for the work included in this submission. Review the submission guidelines for detailed requirements. View published research	NO

Powered by Editorial Manager® and ProduXion Manager® from Aries Systems Corporation

articles from *PLOS ONE* for specific examples.<br><br>This statement is required for submission and **will appear in the published article** if the submission is accepted. Please make sure it is accurate.<br><br>**Unfunded studies**<br>Enter: *The author(s) received no specific funding for this work.*<br><br>**Funded studies**<br>Enter a statement with the following details:<br>• Initials of the authors who received each award<br>• Grant numbers awarded to each author<br>• The full name of each funder<br>• URL of each funder website<br>• Did the sponsors or funders play any role in the study design, data collection and analysis, decision to publish, or preparation of the manuscript?<br>• **NO** - Include this sentence at the end of your statement: *The funders had no role in study design, data collection and analysis, decision to publish, or preparation of the manuscript.*<br>• **YES** - Specify the role(s) played.<br><br>* typeset	
**Competing Interests**<br><br>Use the instructions below to enter a competing interest statement for this submission. On behalf of all authors, disclose any competing interests that could be perceived to bias this work—acknowledging all financial support and any other relevant financial or non-financial competing interests.<br><br>This statement is required for submission and **will appear in the published article** if the submission is accepted. Please make sure it is accurate and that any funding sources listed in your Funding Information later in the submission form are also declared in your Financial Disclosure statement.	NO authors have competing interests

View published research articles from *PLOS ONE* for specific examples.<br><br>**NO authors have competing interests**<br><br>Enter: *The authors have declared that no competing interests exist.*<br><br>**Authors with competing interests**<br><br>Enter competing interest details beginning with this statement:<br><br>*I have read the journal's policy and the authors of this manuscript have the following competing interests: [insert competing interests here]*<br><br>* typeset	
**Ethics Statement**<br><br>Enter an ethics statement for this submission. This statement is required if the study involved:<br><br>• Human participants<br>• Human specimens or tissue<br>• Vertebrate animals or cephalopods<br>• Vertebrate embryos or tissues<br>• Field research<br><br>Write "N/A" if the submission does not require an ethics statement.<br><br>General guidance is provided below. Consult the submission guidelines for detailed instructions. **Make sure that all information entered here is included in the Methods section of the manuscript.**	N/A

**Format for specific study types**<br><br>**Human Subject Research (involving human participants and/or tissue)**<br>• Give the name of the institutional review board or ethics committee that approved the study<br>• Include the approval number and/or a statement indicating approval of this research<br>• Indicate the form of consent obtained (written/oral) or the reason that consent was not obtained (e.g. the data were analyzed anonymously)<br><br>**Animal Research (involving vertebrate animals, embryos or tissues)**<br>• Provide the name of the Institutional Animal Care and Use Committee (IACUC) or other relevant ethics board that reviewed the study protocol, and indicate whether they approved this research or granted a formal waiver of ethical approval<br>• Include an approval number if one was obtained<br>• If the study involved *non-human primates*, add *additional details* about animal welfare and steps taken to ameliorate suffering<br>• If anesthesia, euthanasia, or any kind of animal sacrifice is part of the study, include briefly which substances and/or methods were applied<br><br>**Field Research**<br><br>Include the following details if this study involves the collection of plant, animal, or other materials from a natural setting:<br>• Field permit number<br>• Name of the institution or relevant body that granted permission	
**Data Availability**<br><br>Authors are required to make all data underlying the findings described fully available, without restriction, and from the time of publication. PLOS allows rare exceptions to address legal and ethical concerns. See the PLOS Data Policy and FAQ for detailed information.	Yes - all data are fully available without restriction

A Data Availability Statement describing where the data can be found is required at submission. Your answers to this question constitute the Data Availability Statement and **will be published in the article**, if accepted.<br><br>**Important:** Stating 'data available on request from the author' is not sufficient. If your data are only available upon request, select 'No' for the first question and explain your exceptional situation in the text box.<br><br>Do the authors confirm that all data underlying the findings described in their manuscript are fully available without restriction?	
**Describe where the data may be found in full sentences. If you are copying our sample text, replace any instances of XXX with the appropriate details.**<br><br>• If the data are **held or will be held in a public repository**, include URLs, accession numbers or DOIs. If this information will only be available after acceptance, indicate this by ticking the box below. For example: *All XXX files are available from the XXX database (accession number(s) XXX, XXX.*).<br>• If the data are all contained **within the manuscript and/or Supporting Information files**, enter the following: *All relevant data are within the manuscript and its Supporting Information files.*<br>• If neither of these applies but you are able to provide **details of access elsewhere**, with or without limitations, please do so. For example:<br><br>*Data cannot be shared publicly because of [XXX]. Data are available from the XXX Institutional Data Access / Ethics Committee (contact via XXX) for researchers who meet the criteria for access to confidential data.*<br><br>*The data underlying the results presented in the study are available from (include the name of the third party*	All relevant data are within the manuscript and its Supporting Information files.

and contact information or URL).<br>• This text is appropriate if the data are owned by a third party and authors do not have permission to share the data.<br><br>* typeset	
Additional data availability information:	

Manuscript

Click here to access/download;Manuscript;Manuscript1.docx

Transcriptome analysis of *Tamarix ramosissima* leaves in response to NaCl stress

Yahui Chen[1,2], Guangyu Wang[1],Hongxia Zhang[2,3],Ning Zhang[1*]Jiang Jiang[2*], Zhizhong Song[2,3*]

[1]Department of Forest Resources Management, University of British Columbia, Vancouver, BC V6T 1Z4, Canada

[2]College of Agriculture, Ludong University, Yantai, Shandong, China

[3]Key Laboratory of Molecular Module-Based Breeding of High Yield and Abiotic Resistant Plants in Universities of Shandong (Ludong University), Yantai, Shandong, China

*Correspondence should be addressed to Zhizhong Song wxtba07@163.com. ; Jiang Jiang; ryxiong08@163.com. Ning Zhang； zhangningswing@outlook.com

Subject Category: Genetics and Breeding

Abstract: Halophyte *Tamarix ramosissima.* Lcdcb (*T. ramosissima*) are known as the representative of Tamarix plants that are widely planted in salinized soil. However, molecular mechnisms towards salt tolerance and adaptation are largely rare in *T. ramosissima*. In this study, we carried out RNA-sequence and transcriptome analysis of *T. ramosissima* in response to NaCl stress, and screened differentially expressed genes (DEGs) and further verified by qRT-PCR. Rsults showed that 105702 unigenes were spliced from the raw data of transcriptome sequencing, where 54238 unigenes were retrieved from KEGG, KOG, NR, and Swiss-Prot, and the remaining 51464 unigenes could not be annotated temporarily. After 48 hours of NaCl treatment, the expression levels of 6374 genes in *T. ramosissima* leaves were increased and 5380 genes were decreased. After 168 hours of NaCl stress treatment, the expression levels of 3837 genes were up-regulated and 7808 genes were down-regulated. In particular, 8 highly differentially expressed genes (DEGs) were obtained, which mainly encode WRKY, MYB, and bZIP family transcription factors. In addition, the analysis of the KEGG pathway shows that under NaCl stress, a total of 39 genes in *T. ramosissima* leaves have significant changes in the expression of ROS scavenging mechanisms. After 48 hours of NaCl stress treatment, 21 genes were up-regulated and 18 genes were down-regulated as well as 15 genes were up-regulated and 24 genes were down-regulated after 168h. *T. ramosissima* may adapt to salt stress by enhancing the ROS scavenging mechanism, including SOD, POD, CAT, APX, GPX, GST, and GR. In this study, WRKY, MYB, and bZIP, and other transcription factors may participate in the process of salt tolerance regulation in *T. ramosissima* under NaCl treatment. This study provides gene resources and theoretical basis for further molecular mechanisms of salt tolerance in *T. ramosissima*.

Keywords: *T. ramosissima*; NaCl stress; Transcriptome sequencing; Transcription factor; ROS scavenging mechanisms

1. Introduction

Salinized soil has high salt content and poor soil physical and chemical properties, which seriously hindered the growth and development of plants [1]. Salinized soil contains a lot of Na^+ type salt which can destroy the stability of protein and membrane, and produces osmotic stress and ion poisoning to initiate reactive oxygen ROS (reactive oxygen species) signals in the cell, make dysfunction of the cell, affect the growth of plants, and causes plant death in severe cases [2]. In the past decades, the area of salinized soil has continued to expand due to global human activities a[illegible]limate changes. Favorably, it is becoming a hotspot to carry out

afforestation, restore salinized soil and improve the ecological environment in salinized soil areas.

In recent years, RNA-Seq technology has been widely used in plant science, especially in plant resistance research field. Scholars are using it in halophytes to reveal the molecular mechanisms of plants under salt tolerance [3, 4]. Studies showed that Reactive oxygen species (ROS) will be produced in multiple cell compartments, including chloroplasts [5], mitochondria [6], and peroxisomes [7] under salt stress conditions. A relatively low concentration of ROS are known as an important signal molecule to regulate the normal plant growth and responses to abiotic stresses [8, 9]. However, excessive accumulation of ROS can adversely cause cell oxidative damage [10]. To adapt to ROS damage, higher plants have evolved corresponding regulatory mechanisms to maintain the stability of life activities. Notably, ROS scavenging enzymatic systems, such as superoxide dismutase (SOD), peroxidase (POD), catalase (CAT), ascorbate peroxidase (APX), and glutathione peroxidase (GPX), are prone to play important roles to adapt to undesired abiotic stresses [11]. In addition, plants can also take advantage of the ascorbic acid- reduced glutathione (AsA-GSH) cycle, which contains APX, glutathione reductase (GR) and AsA, and GSH, and so on, to eliminate the damage of ROS [12].

In particular, transcription factors are the most important regulatorsfor plants to respond to various abiotic stresses [13]. In details, reports are focused on the involvement of transcription factors of WRKY [14], bHLH [15], bZIP [16] NAC [17], MYB [18], AP2/ERF [19] under salt stress. In wheat, the WRKY transcription factor gene *GhWRKY34* was induced by salt stress to strengthen the salt tolerance in transgenic *Arabidopsis* and wheat [20]. In *Paspalum notatum*, the

WRKY transcription factor genes are involved in regulating the expression of SOD and related oxidoreductase genes to adapt to salt stress [21]. Notably, *Tamarix* plants have evolved a complex regulatory network for a long time to adapt to the adverse abiotic stresses [22]. *T. hispida* is often used as a biological material to explore the molecular mechanisms towards salt stress tolerance. Overexpression of the *T. hispida ThCOL2* gene can regulate the activity of protective enzymes and reduce the accumulation of O^{2-} and H_2O_2 that enhanced the ROS scavenging abilityand improved the adaptability of transgenic *T. hispida* under salt stress [23]. Overexpression of *ThbZIP1* enhanced the activity of POD and SOD, increased the content of soluble sugar and soluble protein that further improved salt tolerance in transgenic *T. hispida* [24]. *T. ramosissima* usually grows in arid and semi-arid regions with high salt content [25]. Moreover, low concentration (＜100mM) NaCl promoted while high concentration (⩾200mM) NaCl stress inhibitedthe growth of of *T. Ramosissima* [26]. Liu and col[illegible]ues found that *T. ramosissima* exhibited the strongest salt tolerance among 3 *Tamarix* species (*Tamarix gansuensis* H.Z.Zhang, *Tamarix leptostachys* Bunge, *Tamarix ramosissima*. Lcdcb), and*T. ramosissima* was chosen as the representative plants of *Tamarix* plants to further study the molecular mechnisms towards salt tolerance and adaptation.

In this study, we performed high-throughput transcriptome sequencing in *T. ramosissima* under NaCl stressand screened and verified differentially expressed genes (DEGs) at the transcriptional level. This study lays a theoretical basis to reveal molecular mechanisms towards salt tolerance in *Tamarix* plants, and provides gene resource for further variety breeding of salt tolerant plants.

2. Materials and Methods

2.1. Plant materials

T. ramosissima seedlings were provided by the Dongying Experimental Station of Shandong Academy of Forestry Sciences. Experiments were completed at the Key Laboratory of Forest Tree Genetic Breeding and Biotechnology of the Ministry of Education of Nanjing Forestry University from October 2019 to March 2021. 5-month-old *T. ramosissima* seedlings with uniform growth were transferred to a 24-well hydroponic box (size: 40cm*30cm*16cm), supplemented with 1/2 Hoagland nutrient solution, and then placed in a greenhouse that was maintained at 26 (± 2) □ with a relative humidity of 40% to 55% for 1 month after training before treatment.. The culture solution was changed at every 3rd day...

2.2 NaCl treatment

In the control (CK) group, seedlings were suffered with 1/2 Hoagland nutrient solution. In the treatment group, seedlings were cultured in 1/2 Hoagland nutrient solution, supplemented with 200 mM NaCl. 8 plants were used in each group, and 3 times of the experiments were repeated. The culture solution was changed every 3 days. The leaf samples were collected at 0h, 48h, and 168h, respectively, and immediately frozen in liquid nitrogen, and then moved to -80°C refrigerator for storage.

2.3 Transcriptome sequencing

The frozen leaf samples were used for 3-generation high-throughput transcriptome sequencing, using Illumina HiSeq^{TM}4000, inGuangzhou GENE Denovo Company. .The purified PCR products were analyzed by pair-end sequencing (PE150) on the platform according to standard operations, and then fastp was used for quality control, and the original data was filtered and assembled to obtain clean reads [27]. These assembled fragments without N terminal obtained by read overlap are used as the assembled Unigene, and then use Blast2 GO [28] and KOBAS

[29] to obtain Gene Ontology (GO) function and Kyoto Encyclopedia of Genes and Genomes (KEGG) annotation.

2.4 Differential gene screening

DESeq2 software was used to analyze the reads count data to obtain the final correct FDR value [30]. A corrected P value of <0.05 is considered to be significantly enriched. Based on the results of the difference analysis, genes with FDR<0.05 and $|log2FC|>1$ are considered to be significantly different genes.

2.5 Quantitative Real-Time PCR (qRT-PCR) validation of DEGs

Eight putative genes (*Unigene0104732*, *Unigene0028215*, *Unigene0083695*, *Unigene0069097*, *Unigene0090596*, *Unigene0024962*, *Unigene0007135* and *Unigene0088781*) were selected to verify the accuracy of the RNA-Seq results by the qRT-PCR technique. The total RNA was extracted with Omega RNA Extraction kit (Shanghai, China), and then reverse transcribed the 1-strand cDNA by using the PrimerScript™ RT Master Mix kit (TaKaRa, Dalian, China). Specific primers of DEGs are designed via Primer-BLAST server (Supplemental Table 1).. qRT-PCR samples were labelled with PowerUp™ SYBR Green Master mix reagent (Thermo Fisher, China), and then performed on ABI ViiA™ 7 Real-time PCR system (ABI, USA). A total of 3 biological repeats were performed, each with 4 technical repetitions. *Actin* was used as the internal reference gene, and the relative expression level was calculated by the $2^{-\Delta\Delta Ct}$ method.

3 Results

3.1 Sequencing quality analysis

Using IlluminaHiSeq™4000, obtained multiple high-quality bases at 0h, 48h and 168h were obtained in *T. ramosissima* leaves under 200mM NaCl stress, and Q20 reached more than 95% and Q30 reached more than 90%. The percentage of N base number in single-end reads is 0,

and the GC content is above 44% (Table 1), indicating that the quality of the transcriptome sequencing is relatively high, which isreliable for further analysis.

Table 1 Filtered reads quality statistics

Sample	Raw data (bp)	Clean data (bp)	Q20 (%)	Q30 (%)	N (%)	GC (%)
CK1-0h	6341519400	6017997220	5858198656 (97.34%)	5575908163 (92.65%)	125448 (0.00%)	2716858163 (45.15%)
CK2-0h	6216526200	6057604903	5908348667 (97.54%)	5640086542 (93.11%)	138945 (0.00%)	2733246940 (45.12%)
CK3-0h	6627399900	6507140412	6361959382 (97.77%)	6087276423 (93.55%)	119192 (0.00%)	2943970011 (45.24%)
NaCl1-48h	6654895800	6541177895	6461466764 (98.78%)	6275173451 (95.93%)	59608 (0.00%)	2946211482 (45.04%)
NaCl2-48h	6888168900	6782061623	6695451828 (98.72%)	6491216259 (95.71%)	59608 (0.00%)	3048152010 (44.94%)
NaCl3-48h	6720560700	6605897734	6526109306 (98.79%)	6337809692 (95.94%)	54890 (0.00%)	2966230738 (44.90%)
NaCl1-168h	6691086000	6551036697	6475833341 (98.85%)	6299889885 (96.17%)	2997 (0.00%)	2975755051 (45.42%)
NaCl2-168h	6181114500	6032346218	5961590733 (98.83%)	5800920443 (96.16%)	71460 (0.00%)	2741197653 (45.44%)
NaCl3-168h	6396633600	6256461214	6189451116 (98.93%)	6032891080 (96.43%)	74196 (0.00%)	2837598601 (45.35%)

3.2 Unigene basic notes

Results showed that a total of 105,702 Unigenes were spliced in this study. There are 53,385, 46062, 31587, and 36087 Unigenes with gene annotations in the Nr, KEGG, KOG, and SwissProt databases, respectively, and a total of 27,670 Unigenes were simultaneously screened with annotations in the four major databases (Figure 1).

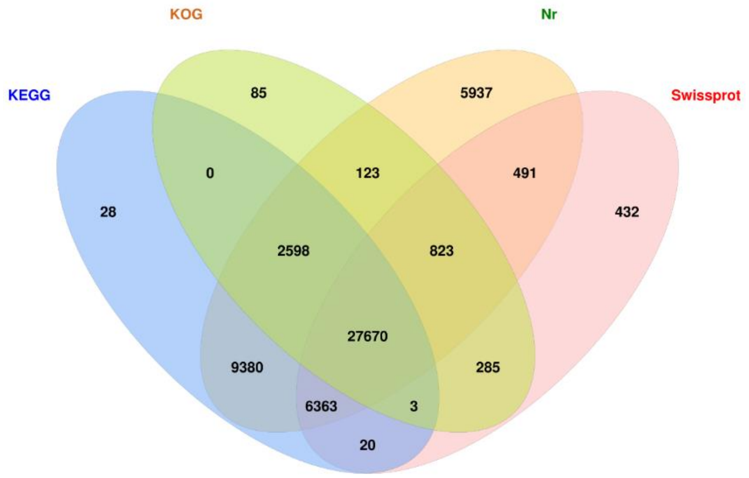

Figure. 1 Annotation diagrams obtained form 4 major databases

3.3 DEGs quantitative analysis

Taking the CK groupas the control, the transcription data of *T. ramosissima* under NaCl stress treatment at 0h, 48h and 168h were compared and analyzed, respectively. DEGs were screened with the standard of FDR<0.05, p<0.05 and |log2FC|>1 after correction. In the CK-0h v.s. NaCl-48h comparationcomparation group, a total of 11,754 gene expression levels were detected, in which 6374 genes were up-regulated and 5380 genes were down-regulated under NaCl treatment. In the CK-0h v.s. NaCl-168h comparationcomparation group, a total of 7768 gene expression changes were detected, inwhich 2542 genes were induced and 5226 genes were reduced under NaCl treatment (Figure 2).

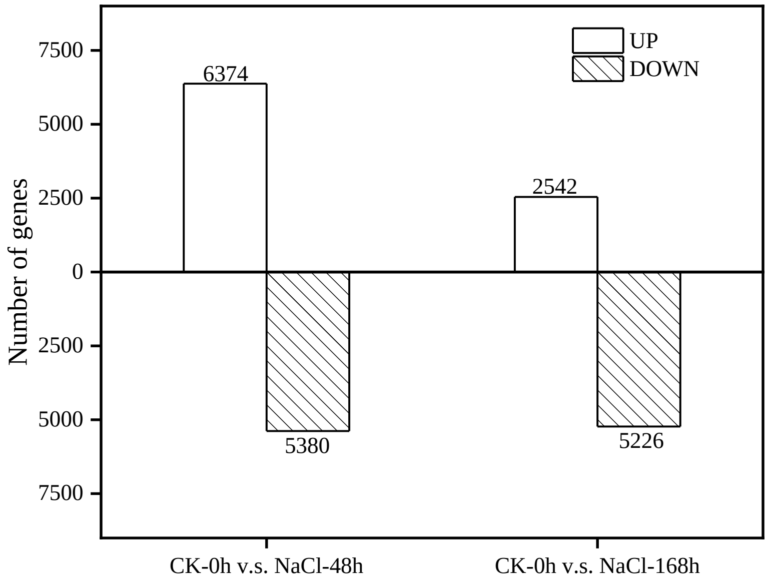

Figure.2 Analysis of differential expression genes

3.4 GO analysis of DEGs

Through GO annotation analysis, the above-mentioned DEGs can be divided into 3 categories: biological processes, cellular components, and molecular functions, and a total of 51 different classification groups were observed (Figure 3). In details, in the CK-0h v.s. NaCl-48h comparationcomparation group, the up-regulated genes were slightly more than the down - regulated genes, while in the CK-0h v.s. NaCl-168h comparation group, the down-regulated

genes were significantly more than the up-regulated genes. In the broad category of biological process, DEGs are mainly concentrated in cellular process, metabolic process, single-organism process and response to stimulus (Table.2). In the molecular function category, DEGs are mainly concentrated in catalytic activity, binding, transporter activity and structural molecule activity (Table.3). Among the major categories of cell components, DEGs are mainly enriched in cell, cell part, organelle and membrane (Table.4). In addition, the number of up-regulated DEGs was significantly lower and the overall number of DEGs decreased, alongside with the time of NaCl treatment. We speculate that *T. ramosissima* leaves may respond to high NaCl stress by inhibiting the expression level of related DEGs at the transcription level.

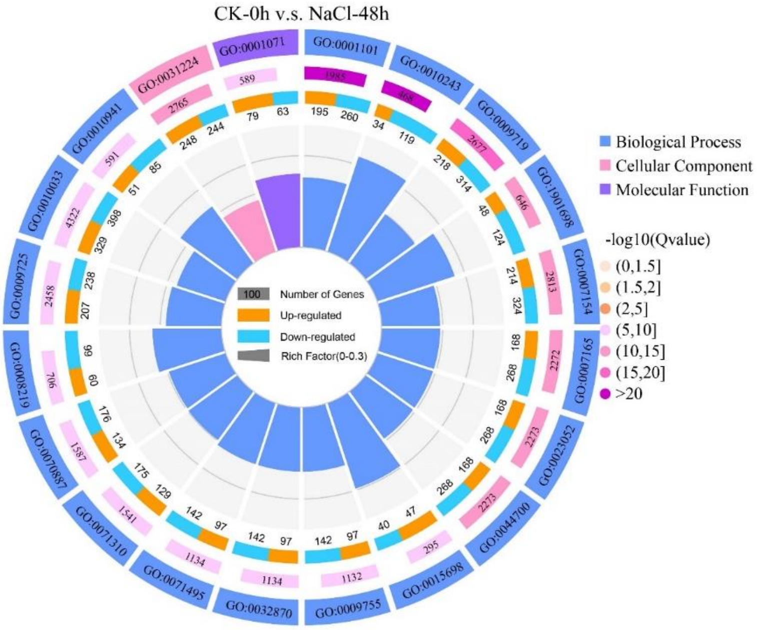

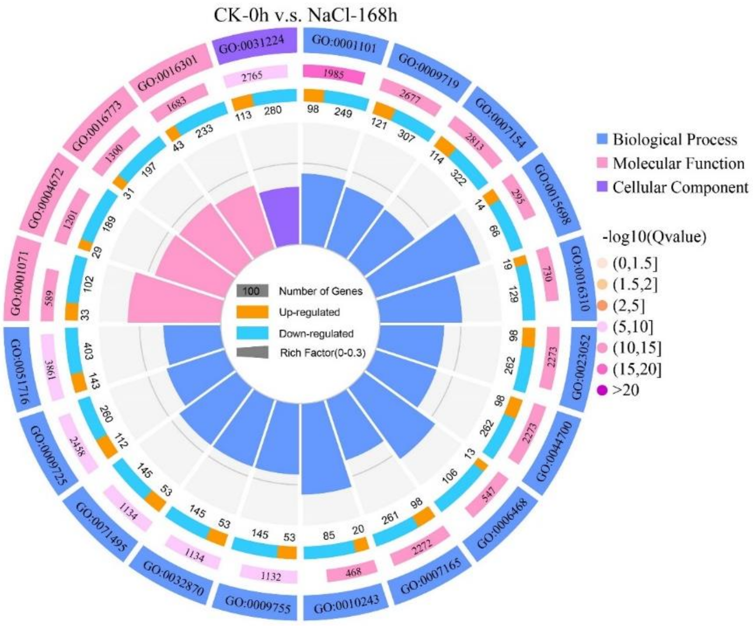

Figure.3 Function analysis of differentially expressed gene GO

Table.2 Annotation analysis of GO functional biological process of DEGs

GO ID	GO Term	CK-0hv.s.NaCl-48h (DEGs)		CK-0hv.s.NaCl-168h (DEGs)	
		Up	Down	Up	Down
GO:0023052	signaling	168	268	98	262
GO:0065007	biological regulation	659	563	269	728
GO:0050789	regulation of biological process	584	514	240	655
GO:0002376	immune system process	73	124	29	130
GO:0032501	multicellular organismal process	398	276	150	394
GO:0050896	response to stimulus	796	875	411	962
GO:0051704	multi-organism process	178	242	95	261
GO:0032502	developmental process	519	373	201	511
GO:0044699	single-organism process	1269	1222	592	1400
GO:0048511	rhythmic process	18	15	15	13
GO:0048519	negative regulation of biological process	90	37	22	70
GO:0048518	positive regulation of biological process	48	61	23	75
GO:0000003	reproduction	222	156	93	226
GO:0040011	locomotion	1	4	1	0
GO:0022414	reproductive process	220	152	91	223
GO:0098754	detoxification	4	0	1	2
GO:0051179	localization	457	436	197	498
GO:0001906	cell killing	0	1	0	4
GO:0040007	growth	83	68	46	102
GO:0022610	biological adhesion	2	4	4	10
GO:0009987	cellular process	1395	1456	659	1638
GO:0008152	metabolic process	1420	1479	659	1630
GO:0071840	cellular component organization or biogenesis	499	417	217	495

Table.3 Annotation Analysis of GO Functional Molecular Function of DEGs

GO ID	GO Term	CK-0hv.s.NaCl-48h	CK-0hv.s.NaCl-168h

		(DEGs)		(DEGs)	
		Up	Down	Up	Down
GO:0001071	nucleic acid binding transcription factor activity	79	63	33	102
GO:0004871	signal transducer activity	11	26	3	30
GO:0005215	transporter activity	146	117	57	152
GO:0016209	antioxidant activity	12	23	6	18
GO:0009055	electron carrier activity	6	9	5	7
GO:0060089	molecular transducer activity	14	19	4	18
GO:0005198	structural molecule activity	119	165	52	130
GO:0003824	catalytic activity	999	1062	458	1212
GO:0098772	molecular function regulator	16	8	8	17
GO:0000988	transcription factor activity, protein binding	1	1	1	2
GO:0005488	binding	939	967	415	1104
GO:0045182	translation regulator activity	0	0	0	0

Table.4 Annotation analysis of GO functional cellular component of DEGs

GO ID	GO Term	CK-0hv.s.NaCl-48h (DEGs)		CK-0hv.s.NaCl-168h (DEGs)	
		Up	Down	Up	Down
GO:0044425	membrane part	311	296	146	333
GO:0016020	membrane	686	732	342	789
GO:0031012	extracellular matrix	1	2	0	2
GO:0044421	extracellular region part	3	3	8	2
GO:0044420	extracellular matrix component	0	1	0	0
GO:0005576	extracellular region	76	97	49	112
GO:0009295	nucleoid	2	2	0	1
GO:0030054	cell junction	193	226	93	248
GO:0099512	supramolecular fiber	2	0	0	5
GO:0019012	virion	0	3	0	2
GO:0044423	virion part	0	3	0	2
GO:0031974	membrane-enclosed lumen	6	12	8	9
GO:0044422	organelle part	502	506	232	497
GO:0032991	macromolecular complex	327	311	119	298
GO:0043226	organelle	1167	1077	595	1180
GO:0005623	cell	1345	1290	667	1430
GO:0044464	cell part	1340	1284	664	1428

3.5 KEGG pathway analysis of DEGs

KEGG pathway analysis showed that 1762 and 1366 DEGs were annotated in the comparation group of CK-0h v.s. NaCl-48h and CK-0h v.s. NaCl-168h, respectively (Figure 4), which directly reflected the changes of gene expression in leaves of *T. ramosissima* under NaCl stress. , Among the top 10 KEGG pathways from the CK-0h v.s. NaCl-48h comparation group, Ribosome (ko03010) annotated to 435 DEGs, accounting for 24.69%, followed by biosynthesis of secondary metabolites (ko01110), phytopathogen interaction (ko04626), phenylpropane

biosynthesis (ko00940), plant hormone signal transduction (ko04075) and plant MAPK signaling pathway (ko04016), respectively, annotated to 416 (23.61%), 88 (4.99%), 69 (3.92%), 69 (3.92%) and 59 3.35% DEGs. Among the top 20 pathways from the CK-0h v.s. NaCl-168h comparation group, the metabolic pathways (ko1100) pathway was annotated to 614 DEGs, accounting for 44.95%, followed by the Biosynthesis of secondary metabolites (ko01110), Ribosomes (ko03010), Oxidative phosphorylation (ko00190)，Plant hormone signal transduction (ko04075) and Plant pathogen interaction (ko04626), respectively, annotated to 351 (25.70%), 296 (21.67%), 104(7.61%)，72 (5.27%) and 63 (4.61%) DEGs. In summary, DEGs are significantly enriched on the KEGG pathway, such as metabolic pathways, biosynthesis of secondary metabolites, and plant hormone signal transduction, in *T. ramosissima* under NaCl treatment.

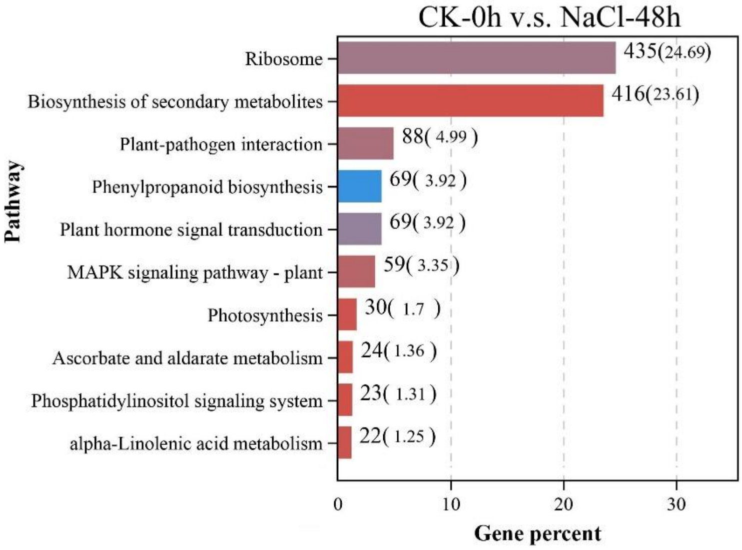

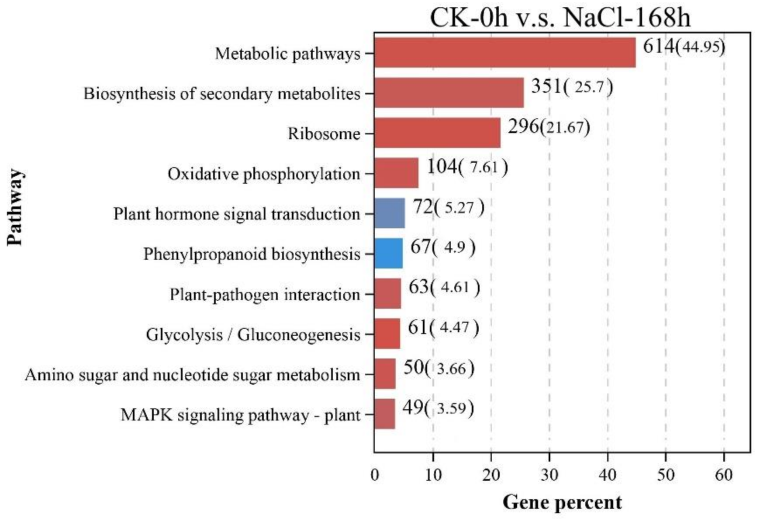

Figure. 4 Top 10 pathway analysis

3.6 Analysis of antioxidant DEGs of *T. ramosissima* leaves under NaCl stress

Results showed the expression of 39 ROS scavenging-related DEGs in the leaves of *T. ramosissima* were changed under salt treatment (Table 5). Notably, there were 21 up-regulated genes and 18 down-regulated genes from the CK-0h v.s. NaCl-48h comparation group. The largest number of up-regulated genes is GST (7), followed by POD (4), CAT (4), APX (3), GR (2) and SOD (1). The largest number of down-regulated gene is GST (8), followed by POD (4), SOD (3), APX (2) and GPX (1). In the CK-0h v.s. NaCl-168h comparation group, there were 15 up-regulated genes and 24 down-regulated genes. In particular, the largest number of up-regulated genes are CAT(3), APX(3) and GST(3), followed by SOD(2), POD(2), and GR(2). The largest number of down-regulated genes is GST(12), followed by POD(6), SOD(2), APX(2), CAT(1) and GPX(1) (Figure 5). These results showed implying that antioxidant mechanism might be initially enhanced in *T. ramosissima* leaves accompanied with corresponding physiological responses to resist NaCl stress during the first 48 hours. However, antioxidant mechanism might be initially inhibited in *T. ramosissima* leaves under long time (168 h) of NaCl treatment, just to adapt to salt stress.

Table.5 Antioxidant DEGs annotated to the KEGG pathway

Pathway	Gene ID	Description	$Log_2FoldChange$	
			CK-0hv.s.NaCl-48h	CK-0hv.s.NaCl-168h
SOD				
ko04146	*Unigene0033269*	SOD4 protein, partial	-1.01	-0.17
	Unigene0049419	superoxide dismutase [Mn]	7.82	9.54
	Unigene0050462	superoxide dismutase	-1.51	-0.60
	Unigene0082550	superoxide dismutase	-0.57	0.26
POD				
ko01100;ko01110; ko00940	*Unigene0009260*	peroxidase 20	-0.46	-0.59
	Unigene0013825	peroxidase	1.79	-1.76
	Unigene0013827	peroxidase	1.17	-0.61
	Unigene0014843	peroxidase	-2.95	-1.80
	Unigene0029752	peroxidase 17	1.51	-0.44
	Unigene0049353	peroxidase 5	-4.98	0.70
	Unigene0086491	peroxidase 52	0.85	-0.74
	Unigene0094375	peroxidase 31	-0.32	1.59
CAT				
ko01100;ko01110; ko01200;ko00630; ko04146;ko04016; ko00380	*Unigene0046159*	catalase isozyme 1	0.58	0.73
	Unigene0046160	catalase, partial	0.77	1.93
	Unigene0087092	leaf catalase	0.07	-0.48
	Unigene0103080	catalase isozyme 1	5.74	12.41
APX				
ko01100;ko00480	*Unigene0008032*	L-ascorbate peroxidase 3	-0.45	-0.24
	Unigene0008033	L-ascorbate peroxidase 3	0.49	0.60
	Unigene0008513	peroxidase domain-containing	0.59	-0.51
	Unigene0048033	cytosolic ascorbate peroxidase	-0.02	0.08
	Unigene0105664	thylakoid ascorbate peroxidase precursor, partial	0.90	1.55
GPX				
ko01100;ko0048; ko00590	*Unigene0035407*	glutathione peroxidase	-0.18	-0.65
GST				
ko01100;ko00480	*Unigene0001041*	glutathione S-transferase	-3.17	-11.56
	Unigene0004890	glutathione S-transferase T1-like	-1.88	-0.68
	Unigene0007072	glutathione S-transferase U17-like	-0.08	0.49

	Unigene0012650	glutathione S-transferase Mu 1-like	13.69	7.20
	Unigene0015109	glutathione S-transferase U8-like	0.11	-0.42
	Unigene0020552	glutathione S-transferase	-0.14	-0.07
	Unigene0041633	microsomal glutathione S-transferase 3-like	0.08	-0.34
	Unigene0048538	glutathione S-transferase U10-like	-3.33	-2.73
	Unigene0056773	glutathione S-transferase	-0.70	0.14
	Unigene0064942	glutathione S-transferase L3	0.28	-0.10
	Unigene0069058	glutathione-S-transferase	0.33	0.92
	Unigene0069060	glutathione S-transferase L3-like	-0.03	-2.16
	Unigene0081745	glutathione S-transferase U10-like	0.05	-0.14
	Unigene0082147	glutathione S-transferase F11-like	2.47	-0.45
	Unigene0098941	glutathione S-transferase U9	-0.17	-5.91
GR				
ko01100;ko00480	*Unigene0075696*	glutathione reductase	0.47	0.12
	Unigene0098587	glutathione reductase-like	8.98	10.52

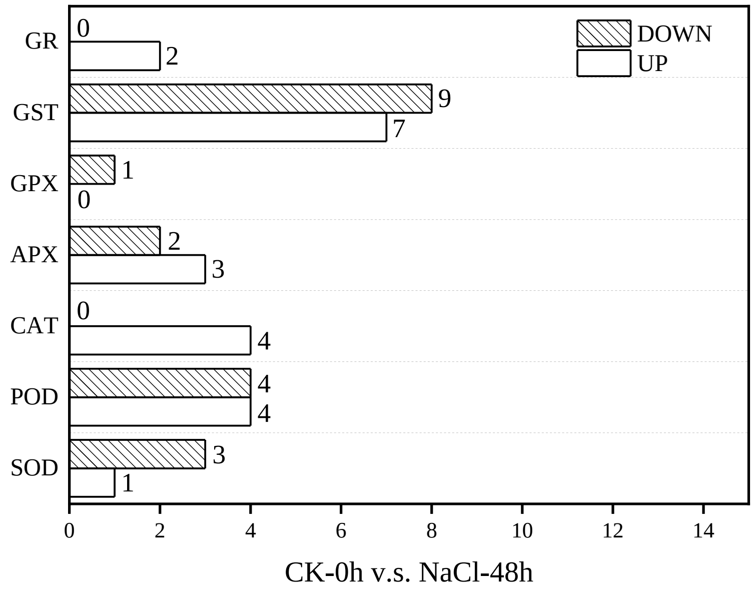

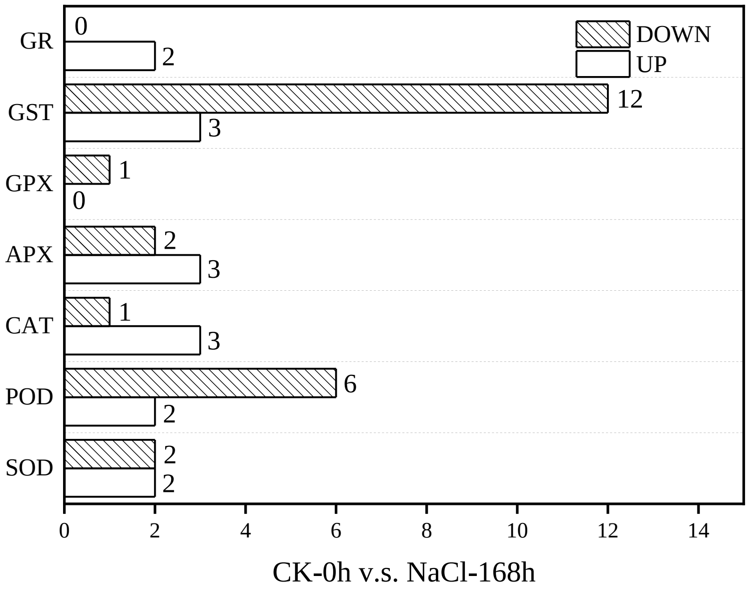

Note: GR：glutathione reductase；GST：glutathione S-transferases；APX：ascorbate peroxidase；GPX：glutathione peroxidase；CAT：Catalases；POD：peroxidase；SOD：Superoxide dismutase

Figure.5 Antioxidant mechanism related genes under NaCl treatment in *T. ramosissima*

3.7 KEGG pathway analysis of key DEGs

Transcription factors play a major role in regulating plant growth and adaptation to adverse environments, including salt stress. In this study, 8 differentiallyexpressed transcription factor genes of WRKY and bZIP were annotated into the KEGG pathway. In particular, 5 WRKY (ko04626 and ko04016) annotated geneswere responsive to NaCl treatment. In particular, the expression levels of *Unigene0010090*, *Unigene0077293* and *Unigene0079542* exhibited a downward trend at 0h, 48h and then an upward trend at 168h. The expression level of *Unigene0014406* and *Unigene0024962* continuously increased until 168h. In addition, 3 bZIP transcription factors were annotated to the KEGG pathway (ko04016, ko01100, ko01110, ko01200, ko01212, ko04146, ko00071, ko00640, ko00410, ko01040, ko00592 and ko04075). The expression level of *Unigene0026888* and *Unigene0008868* exhibited a downward trend 48h and then an upward trend at 168h, while *Unigene0010561* showed a downward trend 48h

and then an upward trend at 168h (Table 6).

Table.6 Gene annotation of transcription factors

Gene ID	Description	Pathway	Log_2 fold change	
			CK-0hv.s.NaCl-48h	CK-0hv.s.NaCl-168h
Unigene0010090	Transcription factor WRKY33	ko04626;ko04016	-0.75	-0.40
Unigene0014406	WRKY DNA-binding protein 27	ko04626;ko04016	0.46	0.79
Unigene0024962	WRKY transcription factor 1	ko04626	0.64	0.69
Unigene0077293	WRKY transcription factor	ko04626;ko04016	-1.96	-2.04
Unigene0079542	WRKY transcription factor 11	ko04626	-0.20.	0.18
Unigene0026888	bZIP4	ko04016	0.67	0.36
Unigene0008868	bZIP2	ko01100;ko01110;ko01200;ko01212;ko04146;ko00071;ko00640;ko00410;ko01040;ko00592	0.6439	-0.011
Unigene0010561	bZIP10	ko04075	-0.8186	-0.38502

3.8 Quantitative Real-Time PCR (qRT-PCR) Validation of Differential Expression

We further randomly selected 8 DEGs involved in salt stress for qRT-PCR verification (Supplemental Table 2). Results showed that the expression level of *Unigene0104732*, *Unigene0083695* and *Unigene0069097* were induced at 48h but reduced at 168h, while genes of *Unigene0090596*, *Unigene0007135* and *Unigene0088781* were decreased at 48h but increased at 168h. Notably, *Unigene0024962* wascontinuously increased while *Unigene0028215* was continuously decreased under NaCl stress. These qRT-PCR verification results are completely consistent with the expression trends observed from the transcriptome sequencing analysis (Supplemental Figure 1). Nonetheless, the transcriptome data obtained in this study is accurate and reliable.

4. Discussion

Tamarix plants have evolved a series of complex mechanisms to resist and adapt to salt stress in the long-term. The response of plants under salt stress is quite complex that needs multiple gene familys and regulation mechnismsinvolved in many biological pathways, including

metabolism, signal transduction, energy production and transportation, ion penetration and transportation [31]. A comprehensive transcriptome analysis of *T. ramosissima* plants helps to reveal the molecular mechanisms of *Tamarix* plants in response to salt stress.

4.1 *T. ramosissima* active oxygen scavenging gene regulation

Plants are usually affected by various unfavorable environmental factors, such as drought, waterlogging, heat damage, cold damage, pests and diseases and heavy metal ions, which resulted in a large amount of ROSaccumulation and relative regulatory mechnisms. Under normal conditions, the production and removal of ROS are in a dynamic equilibrium state and will not affect the growth and development of plants. However, excessive accumulation of ROS can cause oxidative stress, destroy biological macromolecules, biofilms and other structures, and cause cell death in severe cases. In this present study, 39 ROS scavenging related DEGs were significantly regulated by under NaCl stress, implying that ROS scavenging mechanisms was indispensable for *T. ramosissima* plants under NaCl stress.

SOD, POD and CAT are involved in ROS scavenging in plant cells. Under abiotic stresses, expression of SOD, POD and CAT related genes are prone to be up-regulated that furthercontributes to enhance enzyme activitiesto remove ROS, and then ultimately improve plant resistance to abiotic stresses [32]. Notably, the expression level of *TaSOD1.7* gene in leaves increased significantly, and the salt tolerance of transgenic wheat was enhanced [33]. In this study, *T. ramosissima* had 1, 4 and 4 genes related to SOD, POD and CAT activities, respectively, were induced at 48 h. Simultaneously, there are 3 SOD and 4 POD related genes are down-regulated. After 168 hours of NaCl stress, there are still 2 SOD, 2 POD and 3 CAT related genes were up-regulated, while 2 SOD, 6 POD and 1 CAT related genes were down-

regulated. These findings indicate that SOD, POD and CAT enzymes play a roleduring the resistance of *T. ramosissima* to salt stress.

Both APX and GPX are the key enzymes for enzymatically removing ROS, protect cells by catalyzing H_2O_2, and play an important role in plant adversity stress. APX family genes are involved in plant tolerance to drought, heat, salt, oxidation and biological stresses [34]. Overexpression of *PtomtAPX* in transgenic plants possessed stronger ROS scavenging ability and increased plant resistance to abiotic stresses [35]. *NtPHGPx* may maintain the redox balance of the inner membrane of tobacco by removing excess lipid peroxide caused by the accumulation of ROS, and enhance the salt tolerance of tobacco [36]. In addition, the expression of *GPX* gene is quite different under different stresses. The expression of *GPX2* and *GPX4* genes in rice was up-regulated under drought and oxidative stress, but was inhibited under high salt, heat shock and cold stresses [37].In this study, the expression of 3 APX related genes was up-regulated and 2 genes was down-regulated under NaCl treatment. However, only one GPX related gene was down-regulated. We guess that *APX* and *GPX* genes in *T. ramosissima* leaves may respond to salt stress in different ways.

Glutathione S-transferase (GST) are involved in detoxification and antioxidant defense, protecting plants from different abiotic stresses and adversities, and playing multiple roles in plant growth and development [38-40]. In particular, GST was involved in salt stress and GST-related genes are non-sensitive to low and medium NaCl (≤100 mM) concentration, but are sensitive to high NaCl (≥200 mM)stress [38]. Overexpression of *SbGST* in transgenic tobacco plays a crucial role in salt stress tolerance [41]. Meanwhile, *GST* also has negative regulation in response to plant stress. *AtGSTU17* negatively regulates the response of *Arabidopsis* to drought and salt stress, and its lower expression level

or gene knockout improves *Arabidopsis* resistance [42]. Therefore, the way GST responds to abiotic stresses is quite complicated. In this study,3 GST related genes were up-regulated and 8 genes were down-regulated in the leaves at 48 h, and 3 genes was up-regulated and 12 genes were down-regulated at 168 h, implying that there may exist bothpositive and negative regulation of GST in *T. ramosissima* under NaCl stress, and the negative regulation may be the dominant.

GR is one of plant antioxidant enzymes and a flavoprotein oxidoreductase, which exists in eukaryotes and prokaryotes, and play an important role in the elimination of ROS in the process of plant oxidative stress [43].The expression of *Oryza sativa GR3* [44] and *Jatropha JcGR* [45] are all up-regulated under salt stress. Similar to the previous studies, two GR related genes were up-regulated in *T. ramosissima* leaves under NaCl stress. We speculate that GR might be involved in the adaptation of *T. ramosissima* to NaCl stress in a positive direction.

4.2 *T. ramosissima* transcription factor gene regulation

Transcription factors are indispensible for plants to respond to abiotic stresses [13]. WRKY is one of most important transcription factorfamilies, which has been verified to participate in a variety of metabolic processes and plays an important role in the regulation of transcriptional reprogramming related to plant biological and abiotic stress responses [46-48]. In this study, *Unigene0024962*, which encodes a WRKY transcription factor, was up-regulated by NaCl treatment, while *Unigene0079542* was decreased at 48 hbut increased at 168 h, which is consistent with the previous reports in *WRKY* family genes in *soybean* [49] and *Reaumuria trigyna* [50]. The expression of *CaWRKY27* in pepper was down-regulated by NaCl stress [51], which is contrast to our findings. Notably, *Unigene0014406* was continuously induced by NaCl treatment, suggesting that this gene may be inevitably involved in the response of *T.*

ramosissima to salt stress. In addition, bZIP transcription factors play an important regulatory role in plant growth and environmental stress response [52, 53]. The expression of*Unigene0008868*was significantly up-regulated by NaCl treatment, which is consistent with that of Huang's findings in ramie, impling that the bZIP transcription factor plays an important regulatory role in response to salt stress [54]. Moreover, MYB is one of the most diversified transcription factor families in plants in terms of quantity and function, which plays important roles under abiotic stresses [55, 56]. In this study, the expression level of *Unigene0088781* decreased in the first 48 hours and then increased in the long time NaCl treatment, suggesting that this gene needs to be active especially in a specific period under salt stress. Similar findings were also observed in *Medicago sativa* seedlings [57] and *Rosa rugosa* petals [58].

5. Conclusions

The raw data of transcriptome sequencing of *T. ramosissima* leaves in response to NaCl stress was spliced into 105702 Unigenes, and 54238 annotated Unigenes were retrieved in the 4 major functional databases of KEGG, KOG, NR and Swiss-Prot. The expression profiles of DEGs are slightly different between short time (48h)and long time (168) treatments. In particular, ROS scavenging genes and transcription factor encoding genes (including WRKY, MYB and bZIP family) are sensitive to NaCl treatment with distinct regulatory status. This study provides theoretical basis and gene resource for further molecular mechanisms towards salt tolerance in *T. ramosissima.*

Conflicts of Interest

We declare that we do not have any commercial or associative interest that represents a conflict of interest with the work submitted.

Authors' Contributions

Yahui Chen, Guangyu Wang, Hongxia Zhang, Ning Zhang, Jiang Jiang and Zhizhong Song are cofirst authors that contribute equally to this work.

Acknowledgments

We are grateful to Guangyu Wang Professor and his group in the University of British Columbia Forest Science Center for collaborative studies. This work was jointly supported by the following grants: the Agricultural Variety Improvement Project of Shandong Province (2019LZGC009), the National Natural Science Foundations of China (32071612), and the China Scholarship Council (202108320311).

Supplementary Materials

Supplemental Table 1：Sequences of specific primers.

Supplemental Table 2：Randomly select 8 differentially expressed genes.

Supplemental Figure 1: Verfication of DEGs by qRT-PCR.

References

[1] Cheng Y, Yang P, Zhao LH, Priyadarshani SVGN, Zhou Q, Li ZY, et al. Studies on genome size estimation, chromosome number, gametophyte development and plant morphology of salt-tolerant halophyte Suaeda salsa. BMC plant biology. 2019; 19(1): 473. DOI:10.1186/s12870-019-2080-8.

[2] Acosta-Motos J, Ortuño M, Bernal-Vicente A, Diaz-Vivancos P, Sanchez-Blanco M, Hernandez, J. Plant Responses to Salt Stress: Adaptive Mechanisms. Agronomy (Basel). 2017; 7(1): 18. DOI:10.3390/agronomy7010018.

[3] Li SF, Fan CM, Li Y, Zhang JH, Sun JS, Chen YH, et al. Effects of drought and salt-stresses on gene expression in Caragana korshinskii seedlings revealed by RNA-seq. BMC Genomics. 2016; 17(1): 200. DOI:10.1186/s12864-016-2562-0.

[4] Bahieldin A, Atef A, Sabir SM, et al. RNA-Seq analysis of the wild barley (H. spontaneum) leaf transcriptome under salt stress. Comptes rendus. Biologies. 2015; 338(5): 285-297. DOI:10.1016/j.crvi.2015.03.010.

[5] Dietz K, Turkan I, Krieger-Liszkay A. Redox- and Reactive Oxygen Species-Dependent Signaling into and out of the Photosynthesizing Chloroplast.Plant physiology (Bethesda). 2016; 171(3): 1541-1550. DOI:10.1104/pp.16.00375.

[6] Huang SB, Van Aken O, Schwarzländer M, Belt K, et al. The Roles of Mitochondrial Reactive Oxygen Species in Cellular Signaling and Stress Response in Plants. Plant physiology (Bethesda).

2016; 171(3): 1551-1559. DOI:10.1104/pp.16.00166.

[7] Sandalio LM, Romero-Puertas MC. Peroxisomes sense and respond to environmental cues by regulating ROS and RNS signalling networks. Annals of botany. 2015; 116(4): 475-485. DOI:10.1093/aob/mcv074.

[8] Fancy NN, Bahlmann AK, Loake GJ. Nitric oxide function in plant abiotic stress. Plant cell and environment. 2017; 40 (4) : 462-472. DOI:10.1111/pce.12707.

[9] Miller G, Suzuki N, Ciftci-Yilmaz S, Mittler R. Reactive oxygen species homeostasis and signalling during drought and salinity stresses. Plant Cell Environ. 2010; 33(4): 453-467. DOI:10.1111/j.1365-3040.2009.02041.x.

[10] Grant JJ. Loake,G, J. Role of reactive oxygen intermediates and cognate redox signaling in disease resistance. Plant Physiol. 2000; 124(1): 21-29. DOI:10.1104/pp.124.1.21.

[11] Zhang X, Zhang L, Chen Y, Wang S, Fang Y, Zhang X, et al. Genome-wide identification of the SOD gene family and expression analysis under drought and salt stress in barley. Plant growth regulation. 2021; 94(1): 49-60. DOI:10.1007/s10725-021-00695-8.

[12] Gupta M, Cuypers A, Vangronsveld J, Clijsters H. Copper affects the enzymes of the ascorbate-glutathione cycle and its related metabolites in the roots of Phaseolus vulgaris. Physiologia plantarum. 1999; 106(3): 262-267. DOI:10.1034/j.1399-3054.1999.106302.x.

[13] Yao W, Wang S, Zhou B, Jiang T, Li CY. Transgenic poplar overexpressing the endogenous transcription factor *ERF76* gene improves salinity tolerance. Tree physiology. 2016; 36(7): 896-908. DOI：10.1093/treephys/tpw004.

[14] Wang S, Wang J, Yao WJ, Li RH, Jiang JY. Expression patterns of WRKY genes in di-haploid Populus simonii x P. nigra in response to salinity stress revealed by quantitative real-time PCR and RNA sequencing. Plant Cell Rep. 2014; 33: 1687-1696. DOI:10.1007/s00299-014-1647-y.

[15] Chen HC, Hsieh-Feng V, Liao PC, Cheng WH, Liu LY, Yang YW et al. The function of *OsbHLH068* is partially redundant with its homolog, *AtbHLH112*, in the regulation of the salt stress response but has opposite functions to control flowering in Arabidopsis. Plant Mol Biol. 2017; 94(4): 531-548. DOI:10.1007/s11103-017-0624-6.

[16] Yan F, Zhu Y, Zhao Y, Y Wang, Y Liu. De novo transcriptome sequencing and analysis of salt-, alkali-, and drought-responsive genes in Sophora alopecuroides. BMC Genomics. 2020; 21(1): 423. DOI:10.1186/s12864-020-06823-4.

[17] Li M, Chen R, Jiang Q, Sun X, Zhang H, Hu Z. *GmNAC06*, a NAC domain transcription factor enhances salt stress tolerance in soybean. Plant Molecular Biology. 2021; 105: 333-345. DOI:10.1007/s11103-020-01091-y.

[18] Wang Y, Zhang Y, Fan CJ, Wei YC, Zhong CL. Genome-wide analysis of MYB transcription factors and their responses to salt stress in Casuarina equisetifolia. BMC plant biology. 2021; 21(1): 328. DOI:10.1186/s12870-021-03083-6.

[19] Zhang J, Shi S Z, Jiang, Y, Zhong F, Chen YH. Genome-wide investigation of the AP2/ERF superfamily and their expression under salt stress in Chinese willow (*Salix matsudana*). Peer J. 2021; 9(6): e11076. DOI:10.7717/peerj.11076.

[20] Zhou L, Wang NN, Gong SY, Lu R, Li Y, Li XB. Overexpression of a cotton (*Gossypium hirsutum*) WRKY gene, *GhWRKY34*, in Arabidopsis enhances salt-tolerance of the transgenic plants - ScienceDirect. Plant Physiology and Biochemistry. 2015; 96: 311-320. DOI ： 10.1016/j.plaphy.2015.08.016.

[21] Qu XH, Zeng HX. Transcriptome Difference Analysis of *Paspalum notatum* in Response to Salt

Stress. Molecular Plant Breeding(in Chinese). 2019; 17: 3515-3523.

[22] Zhu JK. Abiotic Stress Signaling and Responses in Plants. Cell. 2016; 167(2): 313-324.DOI:10.1016/j.cell.2016.08.029.

[23] Lei X, Tan B, Liu Z et al. *ThCOL2* Improves the Salt Stress Tolerance of *Tamarix hispida*. Frontiers in plant science. 2021; 12: 1-13. DOI:10.3389/fpls.2021.653791.

[24] Wang Y, Gao C, Liang Y, Wang C, Yang C, Liu G. A novel bZIP gene from *Tamarix hispida* mediates physiological responses to salt stress in tobacco plants. Journal of plant physiology.2010; 167(3): 222-230. DOI：10.1016/j.jplph.2009.09.008.

[25] Wilson H, Mycock D, Weiersbye IM. The salt glands of Tamarix usneoides E. Mey. ex Bunge (South African Salt Cedar). International journal of phytoremediation. 2016; 19(6): 587-595. DOI:10.1080/15226514.2016.1244163.

[26] Lu Y, Lei JQ, Zeng FJ, Xu LS, Peng SL, Gao Hh et al. Effects of NaCl treatment on growth and Ecophysiology Characteristics of *Tamarix ramossisma*. Journal of Desert Research (in Chinese). 2014; 34: 1509-1515.

[27] Grabherr MG, Haas BJ, Yassour M, Levin JZ, Thompson DA et al. Full-length transcriptome assembly from RNA-Seq data without a reference genome. Nat Biotechnol. 2011; 29(7): 644-652. DOI:10.1038/nbt.1883.

[28] Conesa A, Gotz S, Garcia-Gomez JM, Terol J, Talon M, Robles M. Blast2GO: a universal tool for annotation, visualization and analysis in functional genomics research. Bioinformatics. 2005; 21(18): 3674-3676. DOI:10.1093/bioinformatics/bti610.

[29] Mao X, Tao C, Olyarchuk, JG, Wei L. Automated genome annotation and pathway identification using the KEGG Orthology (KO) as a controlled vocabulary. Bioinformatics. 2005; 21(19): 3787-3793. DOI:10.2307/1592215

[30] Love MI, Huber W, Anders S. Moderated estimation of fold change and dispersion for RNA-seq data with DESeq2. Genome Biology. 2014; 15(12): 550. DOI:10.1186/s13059-014-0550-8.

[31] Wicker T, Schlagenhauf E, Graner A, Close TJ, Keller B, Stein N. 454 sequencing put to the test using the complex genome of barley. BMC Genomics. 2006, 7(1): 275. DOI：10.1186/1471-2164-7-275.

[32] Noctor G, Reichheld J, Foyer CH. ROS-related redox regulation and signaling in plants. Seminars in cell & developmental biology. 2018; 80: 3-12. DOI:10.1016/j.semcdb.2017.07.013.

[33] Jiang WQ, Yang L, He, YQ, Zhang HT et al. Genome-wide identification and transcriptional expression analysis of superoxide dismutase (SOD) family in wheat (*Triticum aestivum*). Peer J. 2019; 7(2): e8062. DOI:10.7717/peerj.8062.

[34] Lazzarotto F, Teixeira FK, Rosa S, B, Dunand C, Fernandes, CL et al. Ascorbate peroxidase-related (APx-R) is a new heme-containing protein functionally associated with ascorbate peroxidase but evolutionarily divergent. The New phytologist. 2011; 191(1): 234-250. DOI:10.1111/j.1469-8137.2011.03659.x.

[35] Wang B, Cheng ZY, Zhang L, Zhao ZJ, Lu H et al. Tobacco overexpression Populus tomentosa mitochondria ascorbate peroxidase improving stress resistance. Journal of Beijing Forestry University(in Chinese). 2020; 42(7): 33-39.

[36] Guo PC, Yang XN, Mao H, Wang XB, Shen z, Liu D et al. Overexpression and Knockout of NtPHGPx Altered Salt Tolerance of Tobacco Seedlings. Chinese Tobacco Science(in Chinese). 2021; 42(2): 8-14.

[37] Islam T, Manna M, Kaul T, Pandey S, Subramanyam Reddy C et al.Genome-Wide Dissection of

Arabidopsis and Rice for the Identification and Expression Analysis of Glutathione Peroxidases Reveals Their Stress-Specific and Overlapping Response Patterns. Plant Molecular Biology Reporter. 2015; 33(5): 1413-1427. DOI:10.1007/s11105-014-0846-6.

[38] Hao YC, Xu SS, lyu ZF, Wang HW, Sun SL. Comparative Analysis of the Glutathione S-Transferase Gene Family of Four Triticeae Species and Transcriptome Analysis of GST Genes in Common Wheat Responding to Salt Stress. International journal of genomics. 2021, 2021(2), 1-11. DOI:10.1155/2021/6289174.

[39] Horváth E, Bela K, Gallé G, Riyazuddin R, Csomor G et al. Compensation of Mutation in Arabidopsis glutathione transferase (AtGSTU) Genes under Control or Salt Stress Conditions. International journal of molecular sciences. 2020; 21(7):2349. DOI:10.3390/ijms21072349.

[40] Ding, N.; Wang, A.; Zhang, X, Wu Y, Wang R, Cui H, et al. Identification and analysis of glutathione S-transferase gene family in sweet potato reveal divergent GST-mediated networks in aboveground and underground tissues in response to abiotic stresses. BMC Plant Biology. 2017; 17(1): 225. DOI:10.1186/s12870-017-1179-z.

[41] Jha B, Sharma A, Mishra A. Expression of *SbGSTU* (tau class glutathione S-transferase) gene isolated from Salicornia brachiata in tobacco for salt tolerance. Molecular Biology Reports. 2011; 38(7): 4823-4832. DOI:10.1007/s11033-010-0625-x.

[42] Chen J, Jiang H, Hsieh EJ, Chen HY,Chien CT,Hsieh HL et al. Drought and Salt Stress Tolerance of an Arabidopsis Glutathione S-Transferase U17 Knockout Mutant Are Attributed to the Combined Effect of Glutathione and Abscisic Acid. Plant physiology (Bethesda). 2012; 158(1): 340-351. DOI:10.1104/pp.111.181875.

[43] Alscher RG. Biosynthesis and antioxidant function of glutathione in plants. Physiologia plantarum. 1989; 77(3): 457-464. DOI:10.1111/j.1399-3054.1989.tb05667.x.

[44] Wu TM, Lin WR, Kao Y T, Hsu YT, Yeh CH, Hong CY et al. Identification and characterization of a novel chloroplast/mitochondria co-localized glutathione reductase 3 involved in salt stress response in rice. Plant Molecular Biology. 2013; 83(4-5): 379-390. DOI:10.1007/s11103-013-0095-3.

[45] Deng Z, Zhao M, Liu H, Wang YK, Li DJ. Molecular cloning, expression profiles and characterization of a glutathione reductase in *Hevea brasiliensis*. Plant Physiology and Biochemistry. 2015; 96: 53-63. DOI:10.1016/j.plaphy.2015.07.022.

[46] Han, D.; Zhang, Z.; Ding, H.; Chai LJ, Liu W, Li HX et al. Isolation and characterization of *MbWRKY2* gene involved in enhanced drought tolerance in transgenic tobacco. Journal of Plant Interactions. 2018; 13(1): 163-172. DOI:10.1080/17429145.2018.1447698.

[47] Chen F, Hu Y, Vannozzi A, Wu KC, Cai HY, Qin Yet al. The WRKY Transcription Factor Family in Model Plants and Crops. Critical Reviews in Plant Sciences. 2017; 36(4-6): 311-335. DOI:10.1080/07352689.2018.1441103.

[48] Dai Z, Wei M, Zhang BX, Yuan Y, Zhang BF. *VuWRKY*, a group I WRKY gene from Vaccinium uliginosum, confers tolerance to cold and salt stresses in plant. Plant Cell, Tissue and Organ Culture (PCTOC). 2021; 147: 157-168. DOI:10.1007/s11240-021-02115-1.

[49] Wang Y, Jiang L, Chen J, Lei T, Guo C. Overexpression of the alfalfa *WRKY11* gene enhances salt tolerance in soybean. PloS one. 2018;13(2): e192382.DOI:10.1371/journal.pone.0192382.

[50] Du C, Zhao P, Zhang H, Li N, Zheng L, Wang Y. The Reaumuria trigyna transcription factor *RtWRKY1* confers tolerance to salt stress in transgenic Arabidopsis. Journal of Plant Physiology. 2017; 215: 48-58. DOI:10.1016/j.jplph.2017.05.002.

[51] Lin J,Dang FF, Chen YP,Guan DY, He SL. *CaWRKY27* negatively regulates salt and osmotic stress responses in pepper. Plant Physiology and Biochemistry. 2019; 145: 43-51.DOI:10.1016/j.plaphy.2019.08.013.

[52] Li Z, Chao JT, Li XX, Li GB, Liu GS. Systematic Analysis of the bZIP Family in Tobacco and Functional Characterization of *NtbZIP62* Involvement in Salt Stress. Agronomy (Basel). 2021; 11(1): 148. DOI:10.3390/agronomy11010148.

[53] Zhao P, Ye M, Wang RQ, Wang DD, Chen Q. Systematic identification and functional analysis of potato (*Solanum tuberosum* L.) bZIP transcription factors and overexpression of potato bZIP transcription factor *StbZIP-65* enhances salt tolerance. International Journal of Biological Macromolecules. 2020; 161: 155-167. DOI:10.1016/j.ijbiomac.2020.06.032.

[54] Huang C, Zhou J, Jie Y, Xing H, Zhong Y, Yu W et al. A Ramie bZIP Transcription Factor *BnbZIP2* Is Involved in Drought, Salt, and Heavy Metal Stress Response. DNA and cell biology. 2016; 35(12): 776-786. DOI:10.1089/dna.2016.3251.

[55] Shen X, Guo XW, Guo X, Zhao D, Zhao W, Chen J et al. *PacMYBA*, a sweet cherry R2R3-MYB transcription factor, is a positive regulator of salt stress tolerance and pathogen resistance. Plant Physiology and Biochemistry. 2017; 112: 302-311. DOI:10.1016/j.plaphy.2017.01.015.

[56] Wei Q, Zhang F, Sun F, Luo Q, Wang R, Hu R et al. A wheat MYB transcriptional repressor *TaMyb1D* regulates phenylpropanoid metabolism and enhances tolerance to drought and oxidative stresses in transgenic tobacco plants. Plant science (Limerick). 2017; 265: 112-123. DOI:10.1016/j.plantsci.2017.09.020.

[57] Dong W, Gao T, Wang Q, Chen J, Lv J, Song Y. Salinity stress induces epigenetic alterations to the promoter of *MsMYB4* encoding a salt-induced MYB transcription factor. Plant Physiology and Biochemistry. 2020; 155: 709-715. DOI:10.1016/j.plaphy.2020.08.015.

[58] Jiang X, Li S, Ding A, Zhang Z, Liu Q. The Novel Rose MYB Transcription Factor *RhMYB96* Enhances Salt Tolerance in Transgenic *Arabidopsis*. Plant Molecular Biology Reporter 2018; 36: 406-417. DOI:10.1007/s11105-018-1094-y.
