# Supplementary material for: Transcriptome analysis of Tamarix ramosissima leaves in response to NaCl stress
Source: PLoS One. 2022 Mar 31;17(3):e0265653. doi: 10.1371/journal.pone.0265653 (PMC8970367; doi:10.1371/journal.pone.0265653)
Supplement: Attachment — Submitted filename: Response to Reviewers.docx [file pone.0265653.s006.docx]

**Journal:** PLOS ONE

**Title:** Transcriptome analysis of *Tamarix ramosissima* leaves in response to NaCl stress

**Manuscript ID:** PONE-D-21-38993

Dear Edit,

Thank you for giving us the opportunity to resubmit our revised paper for publication in *Plos One*. We greatly appreciate the efforts of you, the associate editor and all reviewers for the constructive comments that have helped shape this manuscript into a better form. We have thoroughly revised the article and addressed all the concerns by either editing the manuscript or clarifying the details in the revised manuscript. Please see the detailed point-by-point response (marked in blue) added below. A 'point-by-point response' to each of the reviewer comments and a marked-up version of the manuscript with the changes highlighted in blue were also uploaded with the clean version of the manuscript files.

Sincerely,

Ning Zhang, Ph.D.

Faculty of Forestry, The University of British Columbia,

Vancouver, British Columbia, Canada

**Additional requirements**

1. When submitting your revision, we need you to address these additional requirements.

Please ensure that your manuscript meets PLOS ONE's style requirements, including those for file naming. The PLOS ONE style templates can be found at
https://journals.plos.org/plosone/s/file?id=wjVg/PLOSOne_formatting_sample_main_body.pdf and
https://journals.plos.org/plosone/s/file?id=ba62/PLOSOne_formatting_sample_title_authors_affiliations.pdf

Response: Thanks for your reminder. The manuscript has been revised to meet PLOS ONE's style requirement.

2. We note that the grant information you provided in the ‘Funding Information’ and ‘Financial Disclosure’ sections do not match.

When you resubmit, please ensure that you provide the correct grant numbers for the awards you received for your study in the ‘Funding Information’ section.

Response: The Funding Information has been revised.

3. Thank you for stating the following in the Acknowledgments Section of your manuscript:
("The authors gratefully acknowledge financial support from the Agricultural Variety Improvement Project of Shandong Province 2019LZGC009, China’s National Science Foundation through grants 32071612 and the China Scholarship Council 202108320311.") We note that you have provided funding information that is not currently declared in your Funding Statement. However, funding information should not appear in the Acknowledgments section or other areas of your manuscript. We will only publish funding information present in the Funding Statement section of the online submission form.

Please remove any funding-related text from the manuscript and let us know how you would like to update your Funding Statement. Currently, your Funding Statement reads as follows:
("The authors gratefully acknowledge financial support from the Agricultural Variety Improvement Project of Shandong Province 2019LZGC009, China’s National Science Foundation through grants 32071612 and the China Scholarship Council 202108320311.")

Please include your amended statements within your cover letter; we will change the online submission form on your behalf.

Response: The funding-related text has been removed from the manuscript.

**In the Funding Statement,**

“The authors received no specific funding for this work.”

We note that you have stated that you will provide repository information for your data at acceptance. Should your manuscript be accepted for publication, we will hold it until you provide the relevant accession numbers or DOIs necessary to access your data. If you wish to make changes to your Data Availability statement, please describe these changes in your cover letter and we will update your Data Availability statement to reflect the information you provide.

Response:

**Data Availability Statement**

All data that support the findings of this study are available. The Illumina raw sequencing data were submitted to the National Center for Biotechnology Information (NCBI) Short Reads Archive (SRA) database under accession number SRP356215.

**Response to Reviewer 1**

Dear Reviewer:

Many thanks for all your valuable comments and suggestions. We carefully revised the manuscript and tried our best to improve it to be a better state to meet the journal’s requirements. The revised manuscript has been submitted to the journal, and looking forward to your further consideration.

**Abstract**

1. Lines:26-34: reorganize and summarize the core contents.

Response: According to your suggestion, we reorganized the abstract and improved greatly in the revised manuscript. Again, thank you for your suggestions.

**Method：**

2. 2.1 and 2.2：Whether the seedlings, Hoagland nutrient solution, and treatment conditions are sterile? If not, the nutrient medium is easy to be polluted, resulting in bacterial treatment of seedlings.

Response: Thanks a lot for your valuable comments. To be honest, the Hoagland nutrient solution used in the study was sterilized before treatment. Before NaCl treatment, the cuttings seedlings were rinsed 3-5 times with sterilized distilled water, and then transferred to the incubator, which was disinfected with 75% alcohol before treatment. Notably, the nutrient solution was changed every third day to avoid media pollution, especially under 26 ℃ in the short-term treatment.

3.Line96 format error: temperature description.line 96 format error: temperature description： “laced in……at 26 (± 2) ℃ .”

Response: Sorry for the incorrect format. It has been revised in the manuscript.

**Results:**

1. Why is the treatment group designed for 2d (48h) and 7d (168h)? The response at the transcriptome level is usually relatively rapid. Did such a long time of NaCl treatment produce phenotypic differences of *T. ramosissima*? IF it is, pictures should be provided.

Response: The purpose of selecting 2d (48h) and 7d (168h) is to observe the changes of gene expression under salt treatment in both the short and the long term. According to previous reports (Song and Su, 2013, Weed Sci; Song et al., 2015, Bio Plantaraum; Song et al., 2016, Sci Horticulturae; Liang et al., 2020 Biomed Res Int; Chen et al., 2021, Int J Genomics), 2d (48h) after stress treatment is a typical time point to monitor the expression levels of responsive genes. While under 7d (168h) treatment, plants exhibited significant physiological phenotype and it is better to discover which DEGs are responsive under salt stress.

2. 3.3 DEGs quantitative analysis

Response: Thank you for your useful suggestions. In the revised manuscript, it was changed into ‘Quantitative expression analysis of DEGs’.

3. 3.4 What is the significance of the analysis of GO notes？Figure2 and Table 2,3,4…Go enrichment analysis can be performed instead of GO annotation analysis statistics…..And what led the authors to this conclusion about “*T. ramosissima* leaves may respond to high NaCl stress by inhibiting the expression level of related DEGs at the transcription level”?

Response: Appreciate your valuable suggestions, and definitely, Go enrichment analysis was performed instead of GO annotation analysis statistics in the revised submission. GO analysis can clearly understand the expression levels of DEGs in three categories: biological processes, cellular components, and molecular functions. According to the distribution of the subclasses of DEGs in each subclass under each major class, it is easy to find which subclasses of DEGs’ expression levels were sensitive to salt stress.

4. 3.6 According to what the 39 genes were concerned and screened from all DEGs? the GO annotation or KEGG or others.

Response: Among all these DEGs, only 39 ROS-related DEGs were specifically found and annotated in the KEGG database.

5. What led the authors to this conclusion about “antioxidant mechanism might be initially inhibited in *T. ramosissima* leaves under long time (168 h) of NaCl treatment, just to adapt to salt stress”? The results are not enough to support the conclusion….. The two diagrams in Figure 5 can be combined.

Response: Frankly speaking, we did analyze the enzymatic activities of SOD, POD and CAT in our previous studies, which were increased at 7d, 15d and 30d. under NaCl stress, compared with the control (Y Chen et al. 2021, 49(15): 142-146, *Jiangsu Agricultural* *Science*, in Chinese), Here, we supplied the enzymatic activities of SOD, POD and CAT in our previous studies, which were increased at 2d (48h) and 7d (168h) in the newly revised manuscript (as shown in Fig. 1).

Figure 1

(Note: The different letters indicate significant differences at the P < 0.05 level

The graph illustrates the changes of enzyme activities in SOD, POD and CAT compared with CK group at 48h and 168h after 200mM NaCl treatment.)

6. 3.7 The title is inappropriate. According to what the 8 genes were concerned and screened from all DEGs? Only these eight? Are there other TFs involved in stress regulation.

Response: According to the transcriptome sequencing of *T. ramosissima* leaves, many transcription factors were discovered. In this study, we did select 8 statically significant DEGs, which were specifically observed and annotated in the KEGG database.

7. 3.8 Unigenes mentioned in 3.6 and 3.7 should be selected and analyzed by qRT-PCR.

Response: Thank you for your valuable comments. In order to verify the reliability of the transcriptome sequencing data, we randomly selected 8 DEGs (including some of the transcription factors screened in this article), according to the description of verification methods in previous publications (He et al., *Forests*. 2020, 11(8); Lu et al., *Biotechnology Bulletin* (in Chinese).2020, 36(12):42-53).

.

8 The authors should detect the related enzyme activities such as POD, SOD, CAT etc. from control and treated *T. ramosissima* to support the conclusion.

Response: Thank you for your kind suggestions. Definitely, we carried out such experiments previously as mentioned above (Chen et al. 2021, 49(15): 142-146, *Jiangsu Agricultural* *Science*, in Chinese).

**Discussion：**

1. The author should condense the discussion part.

Response: We tried our best to rewrite the discussion part. Please see the newly submitted manuscript.

2. Line 284: 4.1 The results are not enough to support the conclusion.

Response: Thanks for the kind reminder. After second thoughts, we revised the content as follows: Enhanced active oxygen scavenging capacity in *T. ramosissima.*

3. Line 290: “Overexpression of *SbGST* in transgenic tobacco plays a crucial role in salt stress tolerance” What is the role?

Response: These findings in tobacco showed that the expression of *SbGST* gene was up-regulated under the stress conditions of salt, cold, drought and salicylic acid. Among them, overexpression of *SbGST* gene in transgenic tobacco promoted seed germination and growth under salt stress. These results confirmed that the expression of *SbGST* genes was up-regulated under different stresses, and the overexpression of *tua* class *SbGST* genes in transgenic tobacco played a crucial role in abiotic stress tolerance. We revised the manuscript in the revised manuscript.

4. Lines 310-313 :4.2What do the authors want to say?

Response: In the 4.2 section of the Discussion part, we want to discuss the differentially expressed responsive transcription factor genes and their possible role under NaCl treatment ins *T. ramosissima.*

5. Lines 322-323 : What led the authors to this conclusion about “that this gene needs to be active especially in a specific period under salt stress”? The results are not enough to support the conclusion.

Response: Many thanks for your valuable comments. After second thought, we have already improved this part in the newly submitted manuscript.

**Others：**

1. Where's the legends of the figures？

Response: Sorry for the careless mistakes. The legends of the figures are supplied in the revised manuscript. Thanks a lot.

2. Writing problems in L149, 152, 160, 210,223,225,255,264,271.

Response: Sorry for our spelling mistakes. All problems have been revised in the newly submitted manuscript.

**To Reviewer 2**

Reviewer #2: A transcriptome analysis of *T. ramosissima* in response to NaCl stress was performed in this study, and some differentially expressed genes (DEGs) were found. However, the methods, results and conclusions should be considered more appropriately and rigorously.

Response:

Response: Appreciate your valuable comments and suggestions. We carefully revised the manuscript and tried our best to improve it to be a better state to meet the journal’s requirements. The revised manuscript has been submitted to the journal, and looking forward to your further consideration

**Introduction Section:**

1. Lines 65-66: *GhWRKY34* is not a wheat gene, please correct it after carefully reading the reference 20.

Response: Thank you so much, and sorry for our typo. *GhWRKY34* is a gene belonging to cotton, which has been revised in the new submission.

**Materials and Methods Section:**

1. In terms of the materials, why choose leaves not roots to study molecular mechanisms towards salt stress?

Response：Thank you for your valuable comments. Frankly, the root growth of *T. ramosissima* is quite slow and rare, especially under salt stress, and the phenotype of roots is not easily altered under the short term of NaCl treatment. While the leaves are the dominant fresh biomass in *T. ramosissima*, and exhibited salt secretion even under the short term of salt stress, which was not observed in roots (Fig. 2). Therefore, we chose the leaves of *T. ramosissima* as the research object to explore responsive DEGs under salt stress.

2. Lines 109-110: The filtering parameters of Fastp should be added. The original data was first filtered to obtain clean reads, then assembled! Not “the original data was filtered and assembled to obtain clean reads.” Please correct it, and add the method as well as software used in assembly.

Response: Thanks a lot for your valuable suggestion. The filtering parameters of Fastp were set in accordance with Chen et al. (2018, Bioinformatics, 34(17):i884-i890), which has been revised in the manuscript.

**Results section:**

Table 1: Please use scientific notation for the data.

Response: According to your valuable suggestion, it has been revised throughout the manuscript.

2. Line 170: “We speculate that *T. ramosissima* leaves may respond to high NaCl stress by inhibiting the expression level of related DEGs at the transcription level.” What exactly does these down-regulated “DEGs” related to?

Response：Thanks for the useful comment. After second thought, we delete this sentence to avoid misunderstanding.

3. Lines 201-202: “Results showed the expression of 39 ROS scavenging-related DEGs in the leaves of *T.ramosissima* were changed under salt treatment.” Please revise it.

Response: According to your suggestion, this sentence was changed into: ‘Results showed that the 39 ROS scavenging-related DEGs were responsive to salt treatment in the leaves of *T. ramosissima*’.

4. Lines 223-224: How many transcription factors in DEGs? Why only WRKY and bZIP were mentioned? What’s the P value and log2FC of these 8 genes?

Response: In total, more than 300 differentially expressed transcription factors genes were screened in this study partially annotated in GO and KEGG databases. In order to verify the reliability of the transcriptome sequencing data, we randomly selected 8 DEGs, which were specifically observed and annotated in the KEGG database, in this study. In addition, the P values of these 8 differentially expressed transcription factor genes were all 0 among the comparisons of CK-0h v.s. NaCl-48h, NaCl-48h vs NaCl-168h and CK-0h v.s. NaCl-168h, indicating a significant high Log_2_ Fold Change of 8 differentially expressed transcription factors. All these details mentioned here were listed in the new Table 1. Appreciate your valuable comments.

Table 1

| Gene ID | Description | Pathway | Log_2_ Fold Change | | |
| --- | --- | --- | --- | --- | --- |
|  |  |  | CK-0h v.s. NaCl-48h | NaCl-48h v.s. NaCl-168h | CK-0h v.s. NaCl-168h |
| *Unigene0010090* | Transcription factor WRKY33 | ko04626;ko04016 | -0.75 | 0.35 | -0.40 |
| *Unigene0014406* | WRKY DNA-binding protein 27 | ko04626;ko04016 | 0.46 | 0.33 | 0.79 |
| *Unigene0024962* | WRKY transcription factor 1 | ko04626 | 0.64 | 0.05 | 0.69 |
| *Unigene0077293* | WRKY transcription factor | ko04626;ko04016 | -1.96 | -0.08 | -2.04 |
| *Unigene0079542* | WRKY transcription factor 11 | ko04626 | -0.20. | 0.38 | 0.18 |
| *Unigene0026888* | bZIP4 | ko04016 | 0.67 | -0.31 | 0.36 |
| *Unigene0008868* | bZIP2 | ko01100;ko01110;ko01200;ko01212;ko04146;ko00071;ko00640;ko00410;ko01040;ko00592 | 0.64 | -0.65 | -0.01 |
| *Unigene0010561* | bZIP10 | ko04075 | -0.82 | 0.43 | -0.39 |

5. Lines 237-238: What kind of genes are Unigene0104732, Unigene0083695 and Unigene0069097? Why randomly choose these three genes instead of choosing genes mentioned above, like WKRY, bZIP?

Response: More than 300 differentially expressed transcription factors genes were screened in this study partially annotated in GO and KEGG databases. In order to verify the reliability of the transcriptome sequencing data, we randomly selected 8 DEGs, which were specifically observed and annotated in the KEGG database, according to the description of verification methods in previous publications (He et al., *Forests*. 2020, 11(8); Lu et al., *Biotechnology Bulletin* (in Chinese).2020, 36(12):42-53).

6. One of the main conclusion, “*T. ramosissima* may adapt to salt stress by enhancing the ROS scavenging mechanism, including SOD, POD, CAT, APX, GPX, GST, and GR.” I don’t think this conclusion is reliable. As the result showed there were some SOD, POD and CAT down-regulated whether after 48 h or 168 h salt treatment. The enzyme activity of SOD, POD, CAT should be measured to see whether ROS scavenging mechanism is enhanced.

Response: Frankly speaking, we did analyze the enzymatic activities of SOD, POD and CAT in our previous studies, which were increased at 7d, 15d and 30d. under NaCl stress, compared with the control (Y Chen et al. 2021, 49(15): 142-146, *Jiangsu Agricultural* *Science*, in Chinese), Here, we supplied the enzymatic activities of SOD, POD and CAT in our previous studies, which were increased at 2d (48h) and 7d (168h) in the newly revised manuscript (as shown in Fig. 1).

Figure 1

(Note: The different letters indicate significant differences at the P < 0.05 level

The graph illustrates the changes of enzyme activities in SOD, POD and CAT compared with CK group at 48h and 168h after 200mM NaCl treatment.)

7. Besides, the article needs careful revision; there are many typos and format error in it.

Response: We would like to thank you again for these valuable comments. We carefully revised the manuscript and tried our best to improve the English level. Hopefully, this revision work will meet your requirement! We need your further help and consideration. Many thanks again！
