# Supplementary material for: Transcriptome analysis of Tamarix ramosissima leaves in response to NaCl stress
Source: PLoS One. 2022 Mar 31;17(3):e0265653. doi: 10.1371/journal.pone.0265653 (PMC8970367; doi:10.1371/journal.pone.0265653)
Supplement: Attachment — Submitted filename: Response to Reviewers.docx [file pone.0265653.s007.docx]

**Journal:** PLOS ONE

**Title:** Transcriptome analysis of *Tamarix ramosissima* leaves in response to NaCl stress

**Manuscript ID:** PONE-D-21-38993

Dear Edit,

Thank you for giving us the opportunity to resubmit our revised paper for publication in *Plos One*. We greatly appreciate the efforts of you, the associate editor and all reviewers for the constructive comments that have helped shape this manuscript into a better form. We have thoroughly revised the article and addressed all the concerns by either editing the manuscript or clarifying the details in the revised manuscript. Please see the detailed point-by-point response (marked in blue) added below. A 'point-by-point response' to each of the reviewer comments and a marked-up version of the manuscript with the changes highlighted in blue were also uploaded with the clean version of the manuscript files.

Sincerely,

Ning Zhang, Ph.D.

Faculty of Forestry, The University of British Columbia,

Vancouver, British Columbia, Canada

**Response to Editor**

1. Please upload a Response to Reviewers letter which should include a point by point response to each of the points made by the Editor and / or Reviewers. (This should be uploaded as a 'Response to Reviewers' file type.)

Response: Thank you for your valuable comments. We have finished editing

2. Please remove your figures/ from within your manuscript file, leaving only the individual TIFF/EPS image files. These will be automatically included in the reviewer’s PDF.

Response: According to your suggestion, We have removed all pictures in the manuscript from the manuscript, and each picture is packaged separately as a PDF file for upload.

**Response to Reviewer 1**

Dear Reviewer:

Many thanks for all your valuable comments and suggestions. We carefully revised the manuscript and tried our best to improve it to be a better state to meet the journal’s requirements. The revised manuscript has been submitted to the journal, and looking forward to your further consideration.

**Abstract**

1. Lines 31-32, Lines 37-38. Please restate the relevant contents of transcription factors

Response: According to your suggestion, we restated all these relevant contents of transcription factors in the revised manuscript. Thank you so much for valuable comments.

2. Line 36 “the enzyme activities of SOD, POD, and CAT were significantly enhanced under NaCl treatment”. The CAT activity had no significant change in Figure2.

Response: Thank you for your useful suggestions. We improved the description of this sentence in the revised manuscript that as following: ‘The enzyme activities of SOD and POD were significantly enhanced under NaCl treatment, but the enzyme activity of CAT was not significantly enhanced’.

3. Line 88 “NaCl stress and screened and verified EGs at the transcriptional level.” Please rewrite this sentence

Response: Sorry for our mistake. In the revised manuscript, it was revised as: ‘NaCl stress and screened and verified DEGs at the transcriptional level’.

**Method：**

1. Line 99 “maintained at 26 ± 2 ℃ (day)() with a relative humidity of 40” Please rewrite this sentence.

Response: Sorry for our mistake. This sentence was revised in the newly submitted manuscript that followed as: ‘maintained at 26 ± 2 ℃ (day) whose relative humidity stays between 40% and 55%’.

2. Line 125 “2.5 Differential gene screening” Please rewrite this little title

Response: According to your valuable suggestion, it has been revised throughout the manuscript as follows: ‘2.5 Screening methods for differentially expressed genes’.

**Results:**

1. Line 127: maybe “corrected P value” and “FDR” is the same parameters?

Response: Many thanks for valuable comments. FDR value means BH corrected *p* value. This has been revised in the manuscript.

2. Lines 209-210: “We speculate that *T*. *ramosissima* leaves may respond to high NaCl stress by reducing the expression level of related DEGs at the transcription level.” This conclusion is inappropriate.

Response: Thank you for your valuable comments. It has been revised in the manuscript that followed as: ‘We speculate that *T*. *ramosissima* leaves may respond to high NaCl stress by affecting the expression level of related DEGs at the transcription level’.

3. Line 228 “3.6 KEGG pathway analysis of DEGs”. Line 275 “3.8 KEGG pathway analysis of key DEGs”. Please rewrite these little titles.

Reply: We rectified these little titles in the newly submitted manuscript as follows: ‘KEGG pathway analysis of DEGs in *T. ramosissima* leaves under NaCl stress’ and ‘3.8 Analysis of transcription factor DEGs in *T. ramosissima* leaves under NaCl stress’.

**Others：**

1. The reason why or how the author chose to pay attention to the eight transcription factors should be explained clearly in the manuscript.

Response: Many thanks for your valuable comments. It has been revised in the manuscript.

2. And the language of the manuscript needs to be carefully revised. Please revise the manuscript carefully.

Response: We would like to thank you again for these valuable comments. We carefully revised the manuscript and tried our best to improve the English level. Hopefully, this revision work will meet your requirement! We need your further help and consideration. Many thanks again!

**To Reviewer 2**

Dear Reviewer:

Response: Appreciate your valuable comments and suggestions. We carefully revised the manuscript and tried our best to improve it to be a better state to meet the journal’s requirements. The revised manuscript has been submitted to the journal, and looking forward to your further consideration

**Introduction Section:**

1. Lines 70-71: “In cotton, the WRKY transcription factor gene *GhWRKY34* was induced by salt stress in transgenic Arabidopsis and wheat.” This sentence is still inaccurate, please correct it after carefully reading the reference.

Response: Sorry for our mistakes. It has been revised in the manuscript that followed as: ‘In cotton, the WRKY transcription factor gene *GhWRKY34* was induced by salt stress in transgenic *Arabidopsis*’.

**Results:**

1. Figure 1：The lower left corner of the Figure 1 was CK-48h? not NaCl-0h? Besides, please add scale bar.

Response: Sorry for our mistake. It has been revised in the manuscript.

2. Figure 2: Please make sure that the data of Figure 2 is unpublished in your previous studies.

Response: We indeed confirm that the data for Figure 2 is not published previously.

3. Table 1: "0.00%" ? Besides, please use scientific notation for the data.

Response: Sorry for our mistake. It has been deleted in the manuscript.

4. Lines 263-265: “However, the antioxidant mechanism might be initially inhibited in *T*. *ramosissima* leaves under a long time (168 h) of NaCl treatment, just to adapt to salt stress.” How do you explain the fact that the antioxidant mechanism was significantly enhanced after 168 h of NaCl treatment (Figure 2).

Response: Sorry for the inaccurate description appeared in the original submission. Under prolonged (168 hours) NaCl treatment, the antioxidant machinery of *T*. *ramosissima* leaves may play a key role in up-regulating genes to adapt to salt stress.

5. As many DEGs were mentioned in 3.7 and 3.8, and some conclusions had been drawn, at least 2-3 genes of the these DEGs should be selected and analyzed by qRT-PCR to support your results and conclusion.

Response: Thank you for your valuable comments. In order to verify the reliability of the transcriptome sequencing data, we randomly selected 8 DEGs (including some of the transcription factors screened in this article), according to the description of verification methods in previous publications [He et al., Forests. 2020, 11(8); Lu et al., Biotechnology Bulletin (in Chinese).2020, 36(12):42-53]. In addition, *Unigene0024962*, as a verified gene, is also a transcription factor annotated to the KEGG Pathway mentioned above.

6. P in “P value” should be italic. All the Figure legends and Table titles should be corrected to meet PLOS ONE's requirements.

Response：According to your valuable suggestion, “*p* value” was written in italic, and all Figure legends and Table titles was corrected in the newly submitted manuscript.

We would like to thank you again for these valuable comments. We carefully revised the manuscript and tried our best to improve the English level. Hopefully, this revision work will meet your requirement! We need your further help and consideration.
